# Supplementary material for: CTRP9 engages AdipoR1 and promotes T cell glycolysis and immunity
Source: EMBO Rep. 2025 Nov 17;26(24):6437–59. doi: 10.1038/s44319-025-00640-0 (PMC12714829; doi:10.1038/s44319-025-00640-0)
Supplement: Supplementary file 1 — Appendix [file 44319_2025_640_MOESM1_ESM.pdf]

Appendix for

CTRP9 engages AdipoR1 and promotes T cell glycolysis and immunity

Table of Contents

Appendix Figure S1 1

Appendix Figure S2 2

Appendix Figure S3 3

Appendix Figure S4 4

Appendix Figure S5 5

Appendix Figure S6 6

Appendix Figure S7 7

Appendix Figure S8 8

Appendix Figure S9 9

Appendix Figure S10 10

Appendix Figure S11 11

Appendix Figure S12 12

Appendix Table S1 13

Appendix Table S2 17

Appendix Figure S1

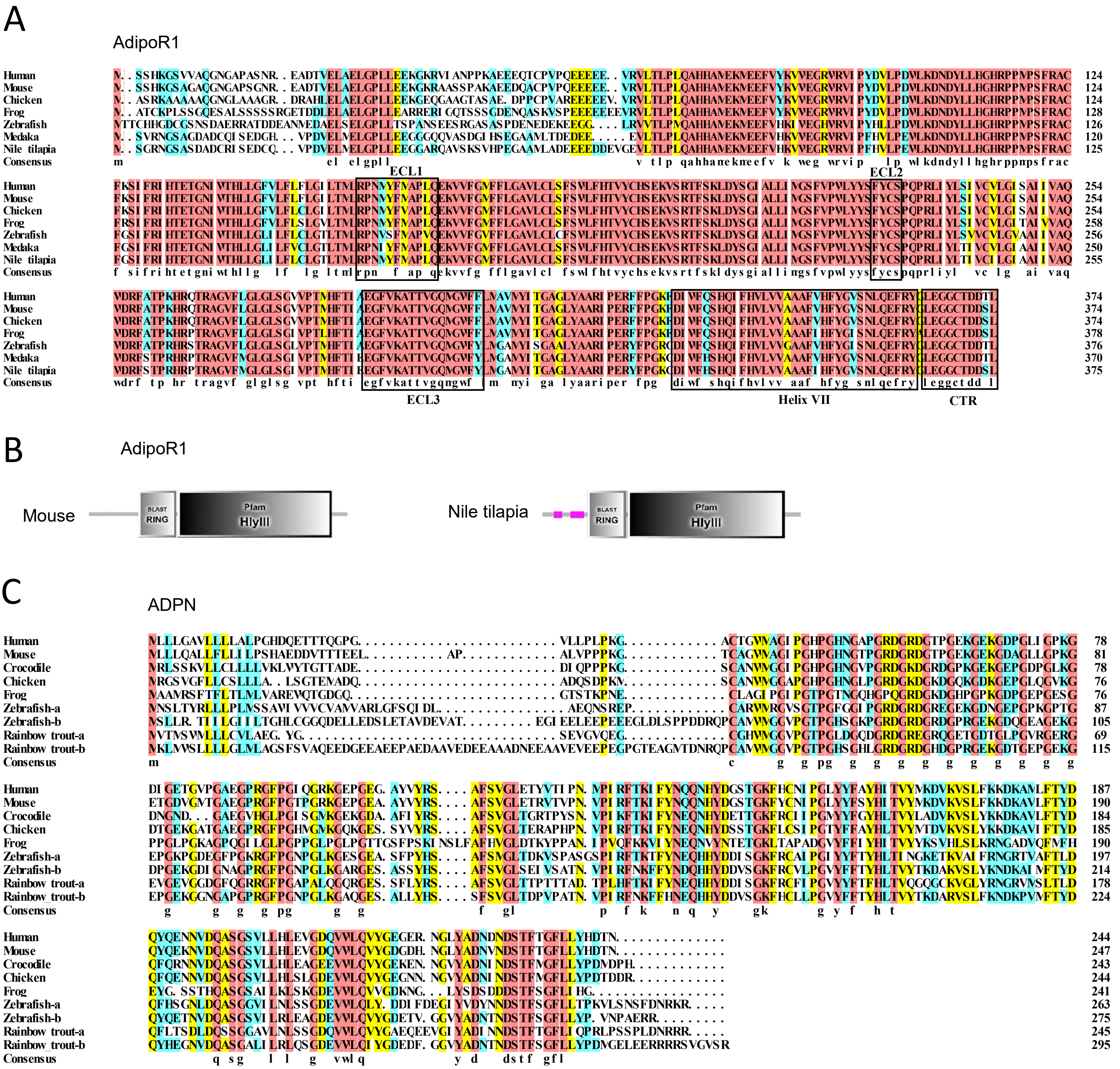

Appendix Figure S1. Sequential analysis of AdipoR1 and ADPN.

(**A**) Multiple sequence alignment of AdipoR1 from tilapia with homologs from other animals. Amino acids with 100% identity are in red, and similar amino acids are in yellow and cyan, respectively. (**B**) Comparison of functional domains of AdipoR1 from mouse and tilapia. (**C**) Multiple sequence alignment of ADPN from other animals. Amino acids with 100% identity are in red, and similar amino acids are in yellow and cyan, respectively.

Appendix Figure S2

A

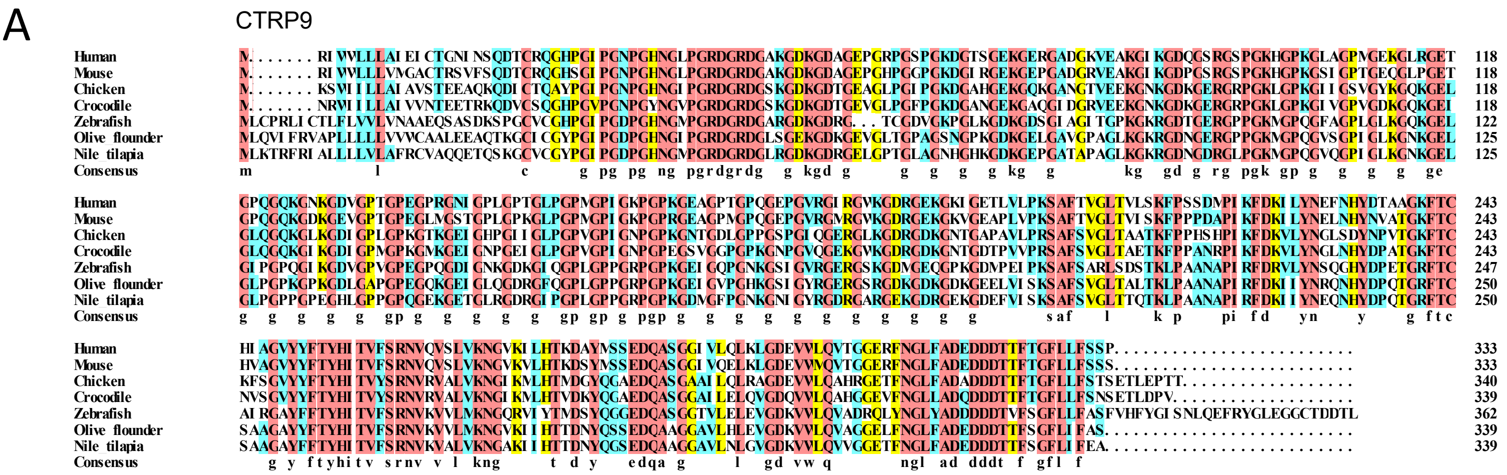

B

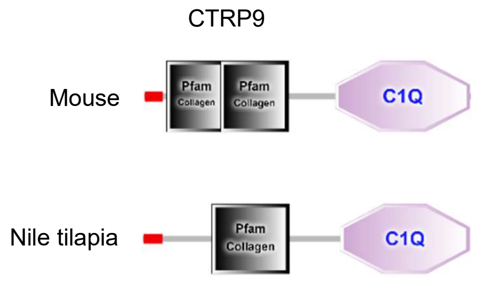

C

Hydrogen bonds			Salt bridges		
AdipoR1	CTRP-9	Dist. (A)	AdipoR1	CTRP-9	Dist. (A)
G91	E325	2.68	R92	D326	2.86
R123	F322	3.44	R92	D326	2.95
S128	L321	3.51	R92	D326	3.90
S128	F322	3.46	R131	D327	3.70
R131	D327	3.70			
R131	E325	3.07			

D

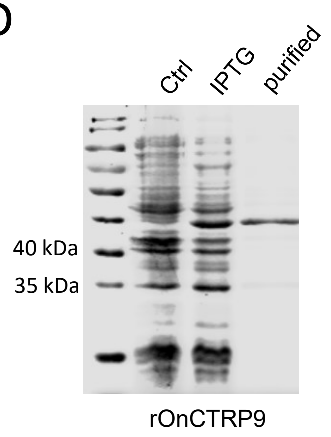

E

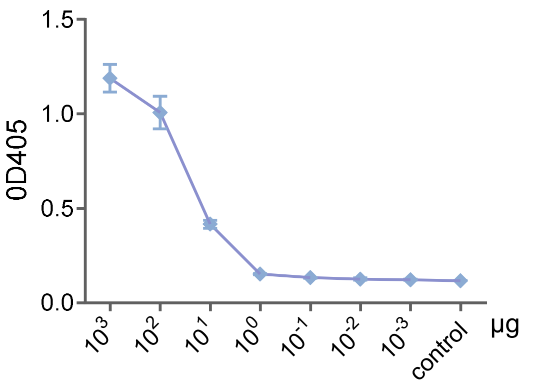

Appendix Figure S2. Sequential properties and recombination of tilapia CTRP9.

(**A**) Multiple sequence alignment of CTRP9 from tilapia with homologs from other animals. Amino acids with 100% identity are in red, and similar amino acids are in yellow and cyan, respectively. (**B**) Comparison of functional domains of CTRP9 from mouse and tilapia. (**C**) Interaction patterns and distance (A= 10^{-10} meters) between docking amino acids in the CTRP-9-AdipoR1 complex. (**D**) SDS-PAGE assay showing the purified recombinant protein of tilapia CTRP9. (**E**) AdipoR1 antibody specificity was detected by ELISA (n=3, n stands for biological replicates). Error bars indicate mean ± SEM.

Appendix Figure S3

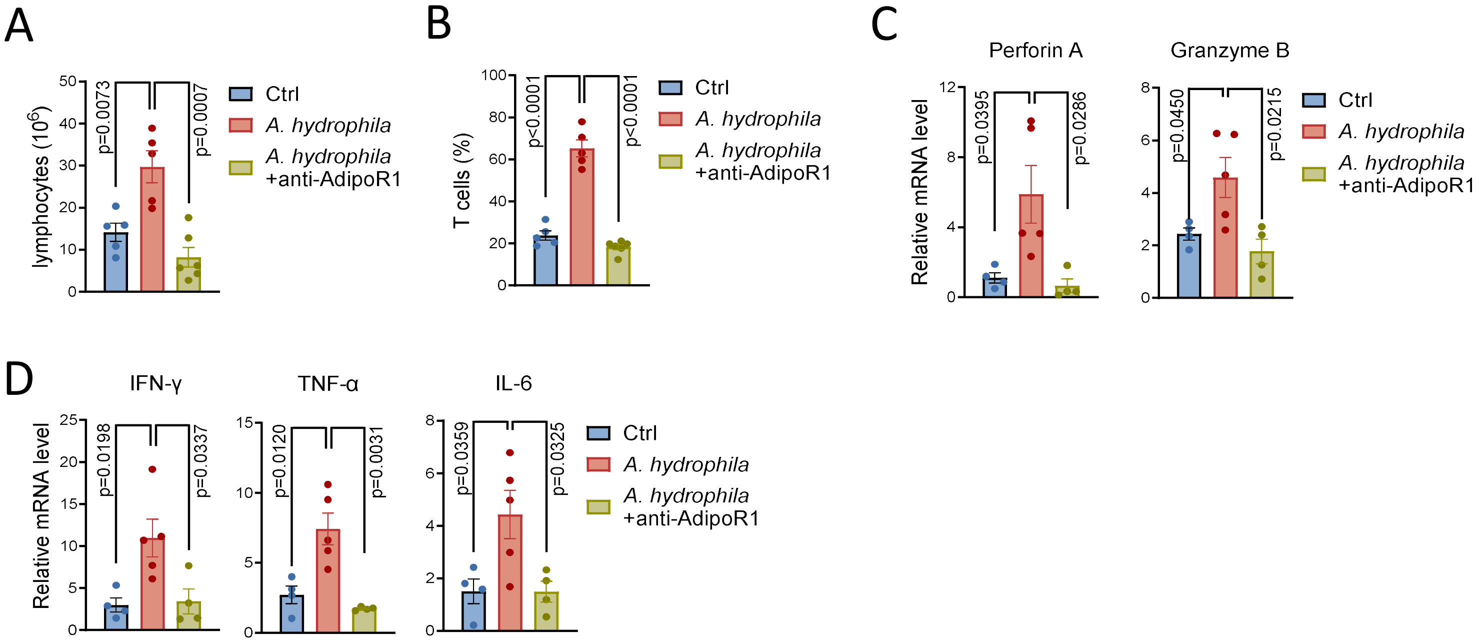

Appendix Figure S3. AdipoR1 plays a key role in T-cell immunity of tilapia. Tilapia individuals infected with *A. hydrophila* were *i.p.* injected with purified rat anti-tilapia AdipoR1 antibody on days 2, 3, 4, and the animals were sacrificed for assay at the indicated time points. (**A-D**) Spleen leukocytes were isolated on 5 dpi. Lymphocytes number (**A**, n=5-6), and percentage of $CD3^{+}$ T cells (**B**, n=5-6) were shown. Relative mRNA levels of indicated genes were examined by qPCR on 5 dpi (**C**, **D**, n=4-5).

Data information: n stands for biological replicates. Error bars indicate mean ± SEM. Significance between the groups was determined by a two-tailed Student's t-test.

Appendix Figure S4

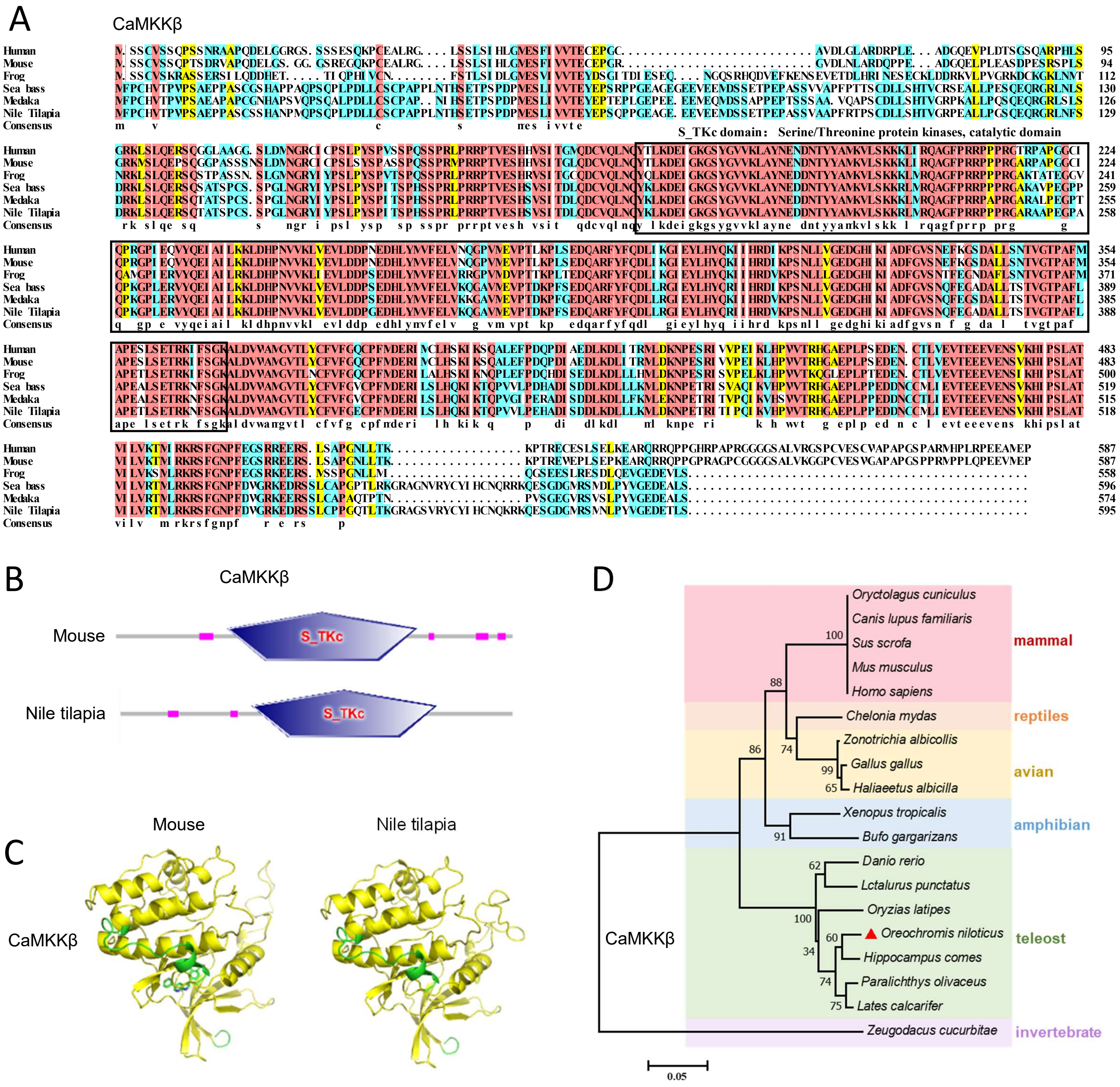

Appendix Figure S4. Sequential and structure properties of tilapia CaMKKβ.

(**A**) Multiple sequence alignment of tilapia CaMKKβwith homologs from other animals. Amino acids with 100% identity are in red, and similar amino acids are in yellow and cyan, respectively. (**B, C**) Comparison of functional domains (**B**) and predicted tertiary structure (**C**) of CaMKKβ from mouse and tilapia. (**D**) Phylogenetic tree constructed with the amino acid sequences of CaMKKβ from the indicated species. The tree was constructed in MEGA7 by using neighbor-joining (NJ) method with 1000 bootstrap replications.

Appendix Figure S5

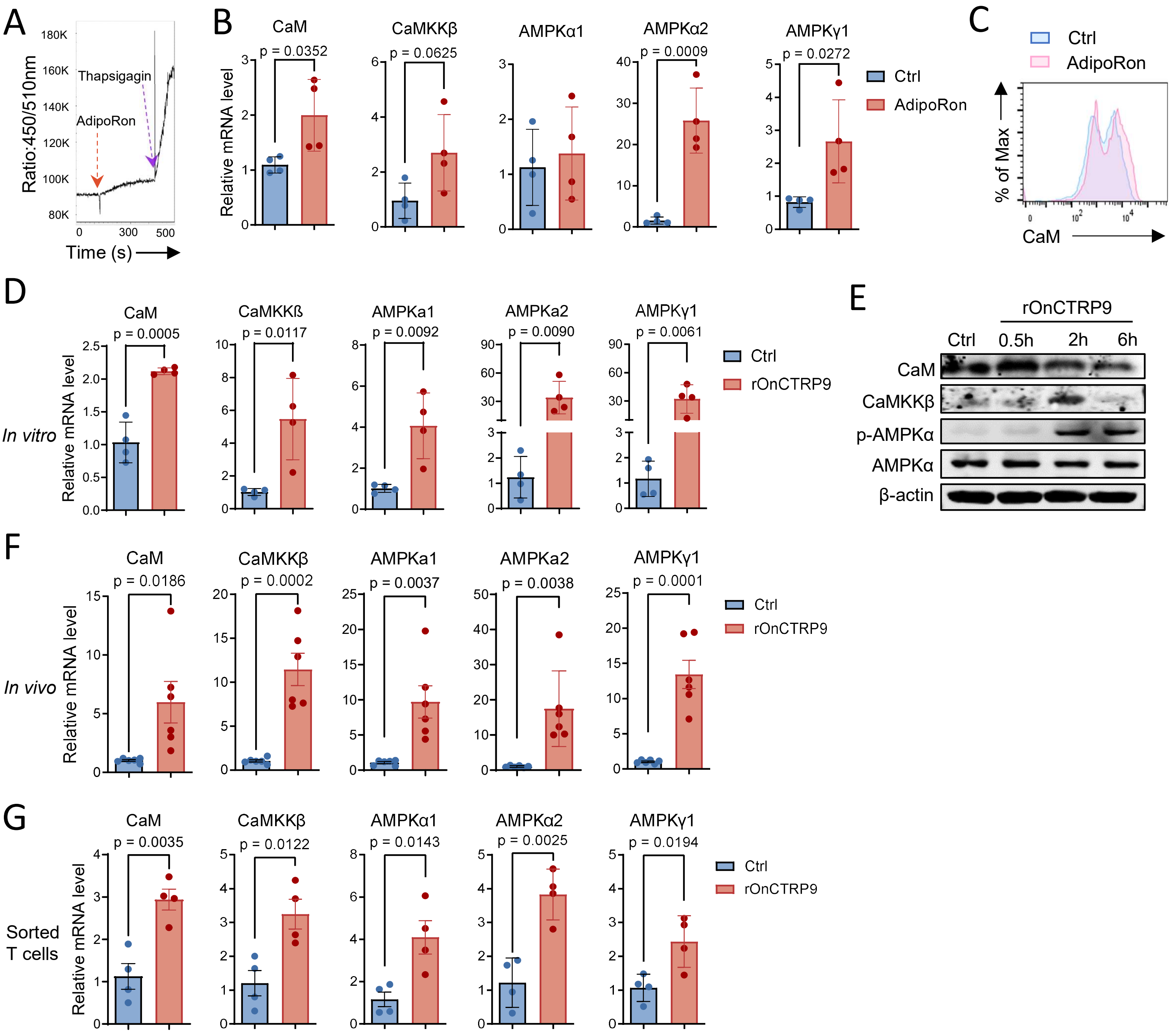

Appendix Figure S5. CTRP9 is an important activator of the Ca^{2+}-CaMKKβ-AMPK axis in tilapia.

(**A**) Ca^{2+} influx of Indo-1–loaded spleen lymphocytes was determined by flow cytometry based on the change in the 450:510 nm ratio after AdipoRon and Thapsigargin stimulation. (**B, C**) Spleen leukocytes were stimulated with AdipoRon. Relative mRNA levels of the indicated molecules were examined by qPCR at 6 h after stimulation (**B**, n=4). Overlaid histograms showing the expression levels of CaM in gated $CD3^{+}$ T cells at 6 h after stimulation (**C**). (**D, E, G**) Spleen leukocytes and sorted T cells were treated with rOnCTRP9. Relative mRNA levels of the indicated molecules were examined in spleen leukocytes (**D**) and sorted T cell (**G**) by qPCR at 6 h after stimulation, n=4. Western blot showing the protein or phosphorylation levels of the indicated molecules in spleen leukocytes after stimulation (**E**). (**F**) Tilapia individuals were *i.p.* injected with or without rOnCTRP9 on days 1 and 2, and the spleen leukocytes were harvested on day 3 for assay. Relative mRNA levels of the indicated molecules were examined by qPCR, n=6.

Data information: n stands for biological replicates. Error bars indicate mean ± SEM. Significance between the groups was determined by a two-tailed Student's t-test.

Appendix Figure S6

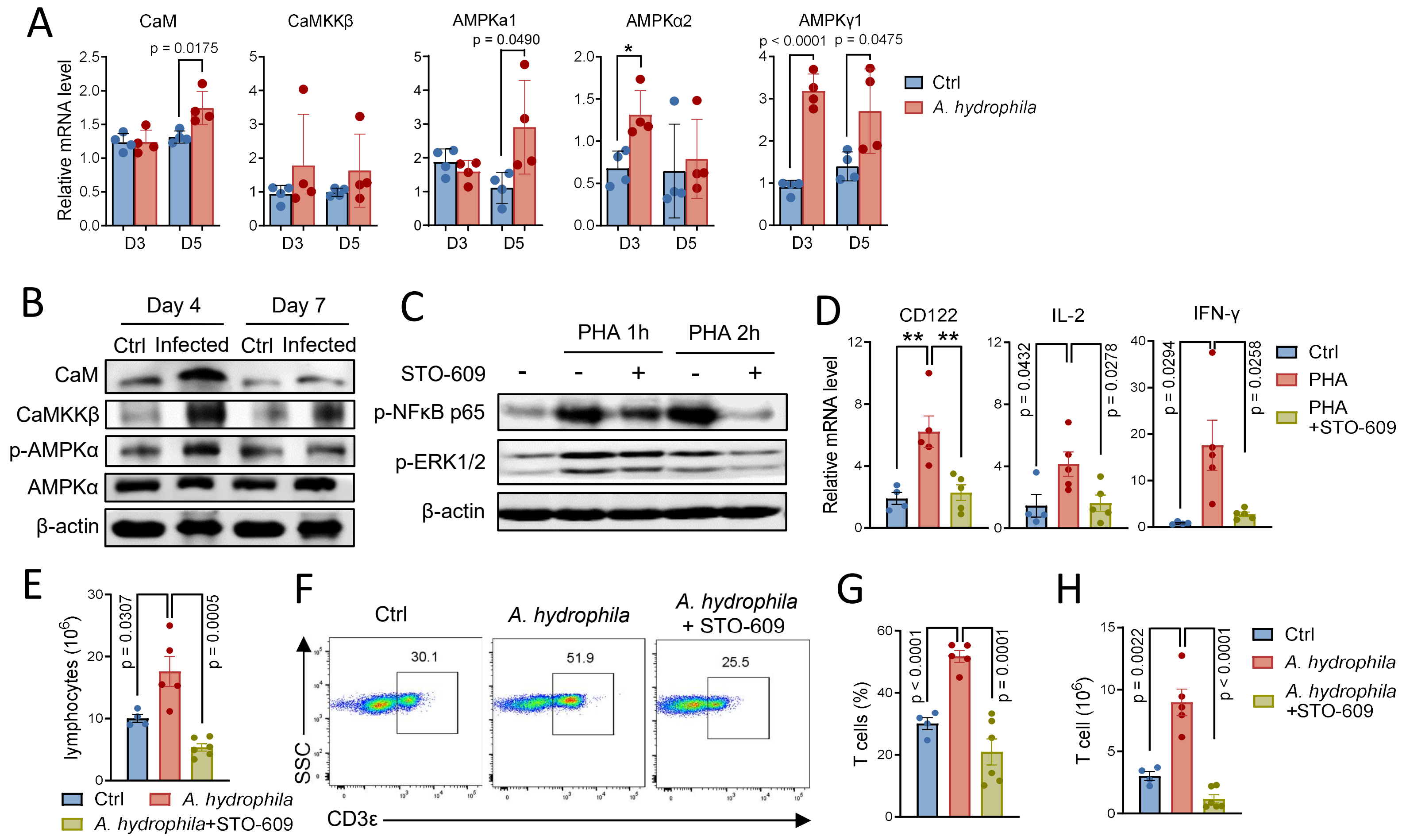

Appendix Figure S6. CaMKKβ-AMPK axis involves in the anti-bacterial immune response of tilapia.

(**A, B**) Tilapia was infected with *A. hydrophila*, (*A*) Relative mRNA levels of indicated molecules in spleen leukocytes were examined by qPCR on 3 and 5 dpi, n=4. (**B**) Western blot assay showing the protein or phosphorylation levels of CaM, CaMKKβ, AMPKα on 4 and 7 dpi. (**C, D**) Spleen leukocytes were treated with PHA and STO-609 or not. Western blot showing the phosphorylation levels of NF-κB p65, ERK1/2 (**C**). Relative mRNA levels of indicated molecules were examined by qPCR at 3 h after stimulation (**D**, n=4-5). (**E-H**) Tilapia individuals that infected with *A. hydrophila* were *i.p.* injected with STO-609 on days 2, 3 and 4, spleen leukocytes were isolated on 5 dpi. Lymphocyte number (**E**), representative FACS plots (**F**), percentage (**G**) and absolute number (**H**) of $CD3^{+}$ T cells were shown, n=4-6. Data information: n stands for biological replicates. Error bars indicate mean ± SEM. Significance between the groups was determined by a two-tailed Student's t-test.

Appendix Figure S7

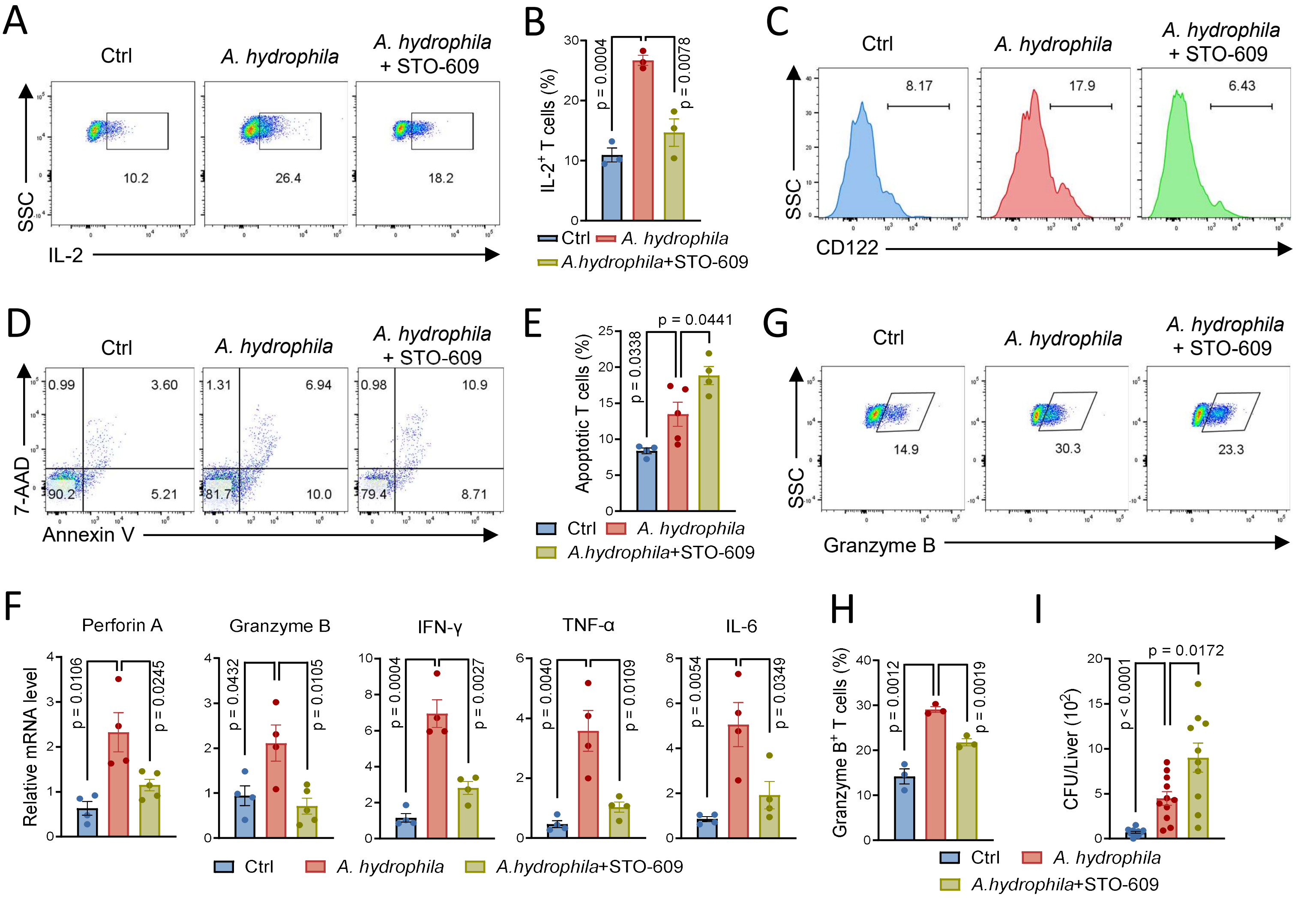

Appendix Figure S7. CaMKKβ is indispensable for T cell immunity in tilapia.

(**A-I**) Tilapia individuals that infected with *A. hydrophila* were *i.p.* injected with STO-609 on days 2, 3 and 4, spleen leukocytes were isolated on 5 dpi. Tilapia individuals were *i.p.* injected with BFA 6 hours before sacrifice. FACS plots (**A**) and bar figure (**B**) showing the percentage of IL-2$^+$ T cells in gated CD3$^+$ T cells, n=3. Histograms showing the expression levels of CD122 in gated CD3$^+$ T cells (**C**). Representative FACS plots (**D**) and bar figure (**E**) showing the 7-AAD and Annexin V staining on gated CD3$^+$ T cell population, n=4-5. Relative mRNA levels of the indicated molecules were examined by qPCR (**F**, n=4-5). Tilapia individuals were *i.p.* injected with BFA 6 hours before sacrifice, and representative FACS plots (**G**) and bar figure (**H**) showing the percentage of granzyme B$^+$ T cells in gated CD3$^+$ T cells T cells, n=3. *A. hydrophila* titers in the liver on 6 dpi (**I**, n=10-11).

Data information: n stands for biological replicates. Error bars indicate mean ± SEM. Significance between the groups was determined by a two-tailed Student's t-test.

Appendix Figure S8

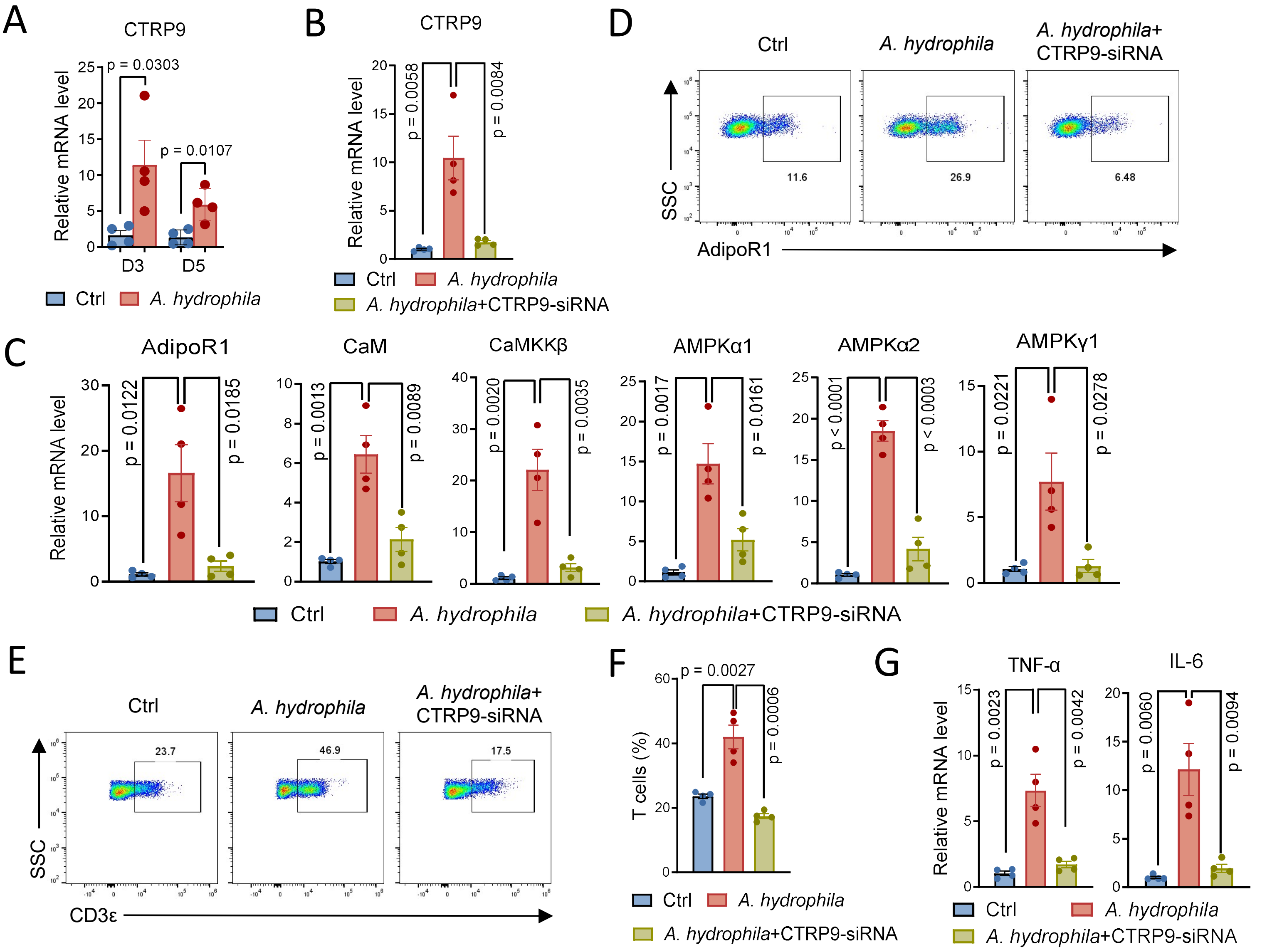

Appendix Figure S8. CTRP9 is important for T cell immunity in tilapia.

(**A**) Tilapia was infected with *A. hydrophila*, relative mRNA levels of *CTRP9* in spleen leukocytes were examined by qPCR on 3 and 5 dpi, n=4. (**B-G**) Tilapia *i.p.* injected with CTRP9-specific or control siRNA were infected with *A. hydrophila*, and spleen leukocytes were harvested on 5 dpi for assay. Relative mRNA levels of the indicated molecules were examined by qPCR (**B, C**, n=4). Representative FACS plots showing the percentage of AdipoR1^{+} T cells in gated lymphocytes (**D**, n=4). Representative FACS plots (**E**) and percentage (**F**) of CD3^{+} T cells were shown, n=4. Relative mRNA levels of *TNF-* and *IL-6* were examined by qPCR (**G**, n=4).

Data information: n stands for biological replicates. Error bars indicate mean ± SEM. Significance between the groups was determined by a two-tailed Student's t-test.

Appendix Figure S9

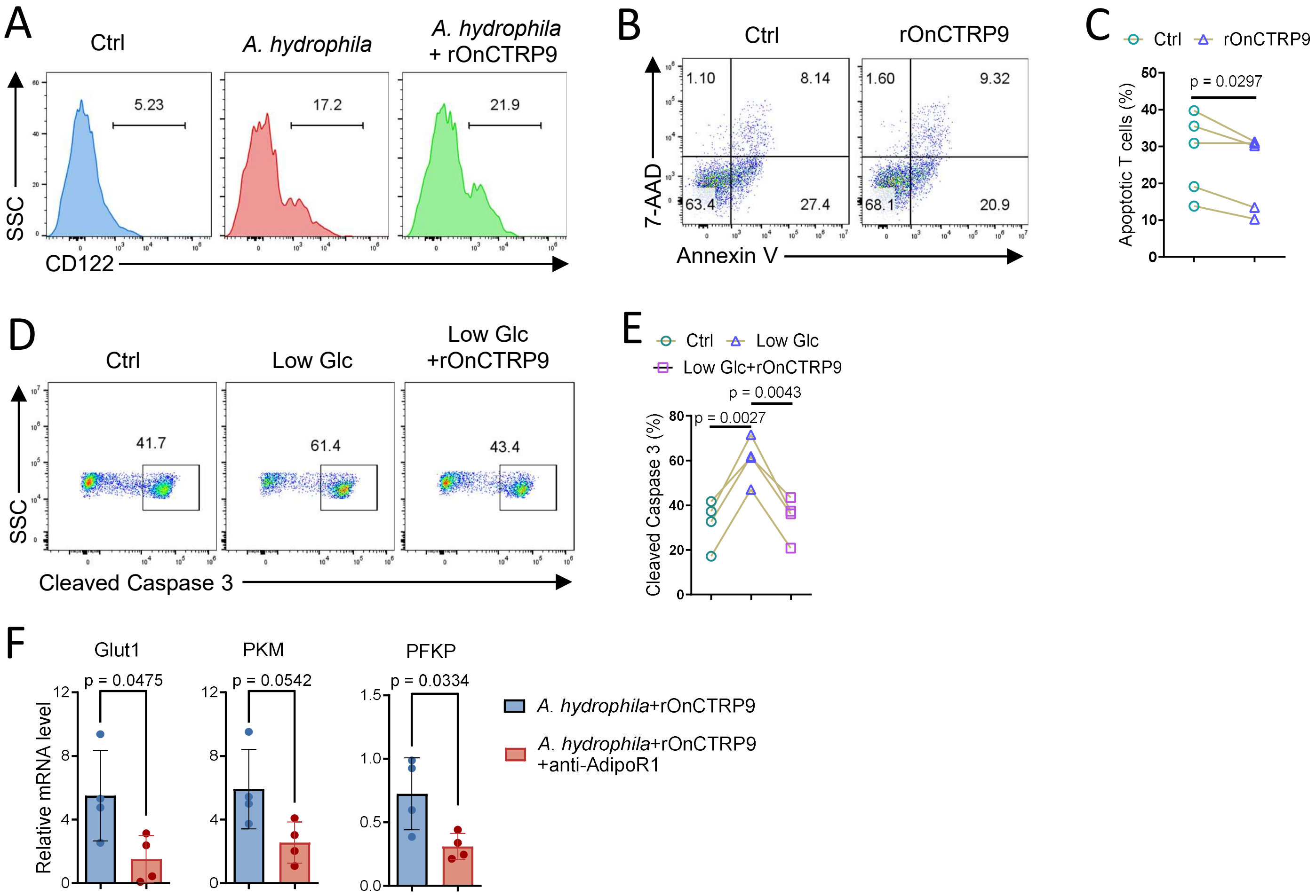

Appendix Figure S9. CTRP9 promotes the T cell glycolysis of tilapia.

(**A**) Tilapia individuals that infected with *A. hydrophila* were *i.p.* injected with or without rOnCTRP9 on days 1, 2, 4, and spleen leukocytes were isolated on 5 dpi. Histograms showing the levels of CD122 in gated CD3$^+$ T cell. (**B, C**) Spleen leukocytes were treated with rOnCTRP9 or not for 12 h. Representative FACS plots (**B**) and statistic figure (**C**) showing the 7-AAD and Annexin V staining on gated CD3$^+$ T cell population. (**D, E**) Spleen leukocytes that cultured in the medium containing normal glucose (4.5g/L) and low glucose (1.125g/L) were treated with rOnCTRP9 or not for 12 h. Representative FACS plots (**D**) and bar figure (**E**) showing the percentage of cleaved caspase 3 in gated CD3$^+$ T cell, n=4. (**F**) Tilapia individuals infected with *A. hydrophila* were *i*.p. injected with or without rat anti-tilapia AdipoR1 antibody on days 1 and 3, or rOnCTRP9 on days 1, 2 and 4. Spleen leukocytes were isolated on 5 dpi. Relative mRNA levels of the *Glut1, PKM, PFKP* were examined by qPCR, n=4.

Data information: n stands for biological replicates. Error bars indicate mean ± SEM. Significance between the groups was determined by a two-tailed Student's t-test.

Appendix Figure S10

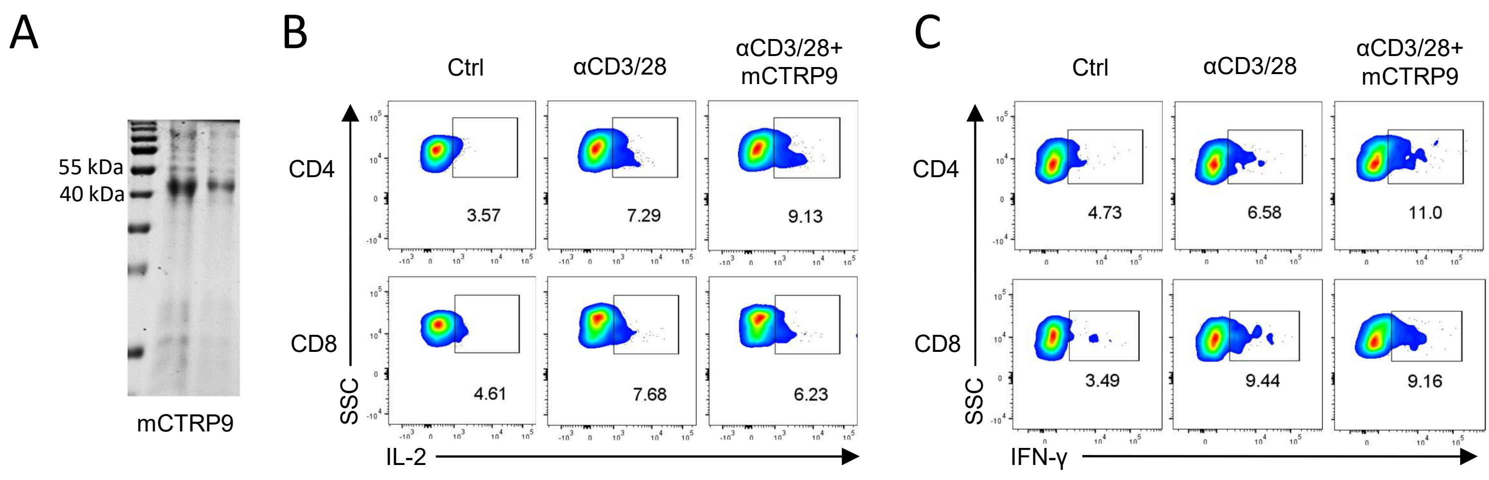

Appendix Figure S10. CTRP9 differentially acts on the cytokine production of CD4⁺ and CD8⁺ T cells in mouse.

(**A**) SDS-PAGE assay showing the purified recombinant mouse CTRP9. (**B, C**) Mouse splenocytes were stimulated with anti-mouse CD3 plus anti-mouse CD28 for 24 h in the presence or absence of mCTRP9, and monensin was used to inhibit cytokines secretion. Representative FACS plots showing the IL-2⁺ cells (**B**) or IFN-γ⁺ cells (**C**) in gated CD4⁺ T cell or CD8⁺ T cell population. The above experiments were repeated for three independent times.

Appendix Figure S11

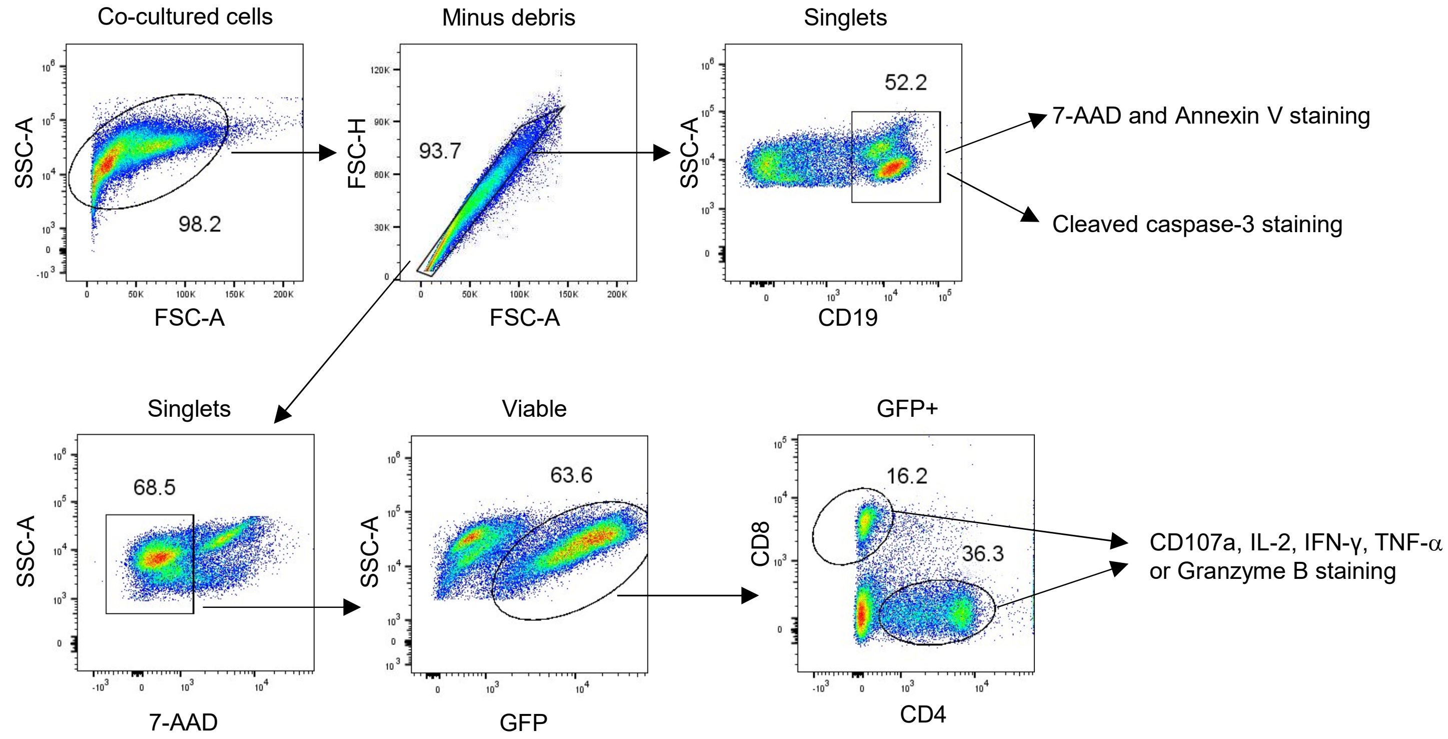

Appendix Figure S11. The flow cytometry strategy for anti-CD19 CAR-T cells and B-cell lymphoma.

CD19^{+} A20 B-cell lymphoma cells were gated from the co-cultured cells for 7-AAD and Annexin V staining or cleaved caspase-3 staining. Viable GFP^{+} anti-CD19 CAR-T cells were gated from the co-cultured cells to identify CD4^{+} or CD8^{+} subpopulation, and then for the CD107a, IL-2, IFN-γ, TNF-α or Granzyme B staining.

Appendix Figure S12

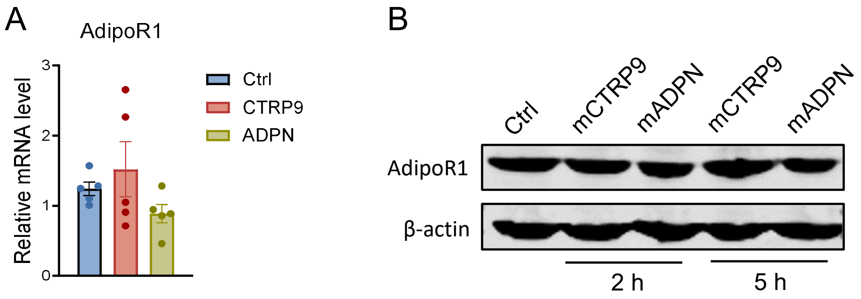

Appendix Figure S12. The expression of mouse AdipoR1 upon treatment with CTRP9 or ADPN. Mouse splenocytes were stimulated with mCTRP9 or mADPN. (**A**) Relative mRNA level of *AdipoR1* were examined by qPCR at 5 h, n=5, n stands for biological replicates. (**B**) Western blot showing protein levels of AdipoR1 at 2 h or 5 h.

Appendix Table S1. Information of genes used for sequence, phylogeny and structure analysis in present study

Source	Accession No.	Gene name	Species	Application
GenBank	XP_003441547.1	AdipoR1	*Oreochromis niloticus*	DP, MSA, 3D, PA, GI
GenBank	NP_001292998.1	AdipoR1	*Mus musculus*	DP, MSA, 3D, PA, GI
GenBank	NP_001277482.1	AdipoR1	*Homo sapiens*	MSA, PA, GI
GenBank	NP_001026198.1	AdipoR1	*Gallus gallus*	MSA, PA, GI
GenBank	XP_004069161.1	AdipoR1	*Oryzias latipes*	MSA, PA, GI
GenBank	NP_001007928.1	AdipoR1	*Xenopus tropicalis*	MSA, PA, GI
GenBank	NP_001314683.1	AdipoR1	*Danio rerio*	MSA, PA, GI
GenBank	XP_019942622.1	AdipoR1	*Paralichthys olivaceus*	PA
GenBank	XP_011439974.1	AdipoR1	*Crassostrea gigas*	PA
GenBank	NP_651061.1	AdipoR1	*Drosophila melanogaster*	PA
GenBank	NP_001007194.1	AdipoR1	*Sus scrofa*	PA
GenBank	XP_018554599.1	AdipoR1	*Lates calcarifer*	PA
GenBank	XP_019736837.1	AdipoR1	*Hippocampus comes*	PA
GenBank	XP_044143219.1	AdipoR1	*Bufo gargarizans*	PA
GenBank	XP_009919418.1	AdipoR1	*Haliaeetus albicilla*	PA
GenBank	XP_002717631.1	AdipoR1	*Oryctolagus cuniculus*	PA
GenBank	XP_005451408.1	CaMKKβ	*Oreochromis niloticus*	DP, MSA, 3D, PA
GenBank	NP_001186605.1	CaMKKβ	*Mus musculus*	DP, MSA, 3D, PA
GenBank	NP_001257414.1	CaMKKβ	*Homo sapiens*	MSA, PA
GenBank	XP_018526027.1	CaMKKβ	*Lates calcarifer*	MSA, PA
GenBank	XP_002937747.1	CaMKKβ	*Xenopus tropicalis*	MSA, PA
GenBank	XP_004075019.1	CaMKKβ	*Oryzias latipes*	MSA, PA
GenBank	XP_021324898.1	CaMKKβ	*Danio rerio*	PA
GenBank	XP_020928685.1	CaMKKβ	*Sus scrofa*	PA
GenBank	XP_025011297.1	CaMKKβ	*Gallus gallus*	PA
GenBank	XP_019938187.1	CaMKKβ	*Paralichthys olivaceus*	PA
GenBank	XP_028898424.1	CaMKKβ	*Zeugodacus cucurbitae*	PA
GenBank	XP_017347702.1	CaMKKβ	*Lctalurus punctatus*	PA
GenBank	XP_019738138.1	CaMKKβ	*Hippocampus comes*	PA
GenBank	XP_044131686.1	CaMKKβ	*Bufo gargarizans*	PA
GenBank	XP_007058349.1	CaMKKβ	*Chelonia mydas*	PA
GenBank	XP_009913518.1	CaMKKβ	*Haliaeetus albicilla*	PA
GenBank	XP_026651985.1	CaMKKβ	*Zonotrichia albicollis*	PA
GenBank	XP_017205068.1	CaMKKβ	*Oryctolagus cuniculus*	PA
GenBank	XP_038292530.1	CaMKKβ	*Canis lupus familiaris*	PA
GenBank	NP_001038890.1	ADPNb	*Danio rerio*	MSA, 3D, GI
GenBank	NP_033735.3	ADPN	*Mus musculus*	MSA, 3D, GI
GenBank	NP_001171271.1	ADPN	*Homo sapiens*	MSA, GI
GenBank	NP_001373470.1	ADPNa	*Danio rerio*	MSA, GI
GenBank	NP_996874.1	ADPN	*Gallus gallus*	MSA, GI
GenBank	XP_006028143.1	ADPN	*Alligator sinensis*	MSA, GI
GenBank	NP_001005793.1	ADPN	*Xenopus tropicalis*	MSA, GI
GenBank	XP_036790926.1	ADPNa	*Oncorhynchus mykiss*	MSA, GI

GenBank	XP_021466932.1	ADPNb	*Oncorhynchus mykiss*	MSA, GI
GenBank	XP_005471911.1	CTRP9	*Oreochromis niloticus*	DP, MSA, 3D,
GenBank	NP_898998.2	CTRP9	*Mus musculus*	DP, MSA, 3D,
GenBank	NP_001290066.1	CTRP9	*Homo sapiens*	MSA
GenBank	XP_015134645.2	CTRP9	*Gallus gallus*	MSA
GenBank	XP_006017705.1	CTRP9	*Alligator sinensis*	MSA
GenBank	NP_001107934.1	CTRP9	*Danio rerio*	MSA
GenBank	XP_019942939.1	CTRP9	*Paralichthys olivaceus*	MSA
GenBank	NP_001362292.1	AdipoR2	*Homo sapiens*	GI
GenBank	XP_030111434.1	AdipoR2	*Mus musculus*	GI
GenBank	NP_001007855.1	AdipoR2	*Gallus gallus*	GI
GenBank	XP_031754470.1	AdipoR2	*Xenopus tropicalis*	GI
GenBank	XP_025067147.1	AdipoR1	*Alligator sinensis*	GI
GenBank	XP_014373370.1	AdipoR2	*Alligator sinensis*	GI
GenBank	XM_025899627.1	AdipoR2	*Oreochromis niloticus*	GI
GenBank	JAQSNX010003226.1	AdipoR1	*Oreochromis grahami*	GI
GenBank	JAQSNX010002709.1	AdipoR2	*Oreochromis grahami*	GI
GenBank	XM_039612649.1	AdipoR1	*Oreochromis aureus*	GI
GenBank	XM_031726544.2	AdipoR2	*Oreochromis aureus*	GI
GenBank	VSJB01164303.1	AdipoR1	*Oreochromis spilurus*	GI
GenBank	VSJB01048971.1	AdipoR2	*Oreochromis spilurus*	GI
GenBank	VUUZ01027956.1	AdipoR1	*Oreochromis malagarasi*	GI
GenBank	VUUZ01025704.1	AdipoR2	*Oreochromis malagarasi*	GI
GenBank	VVHC01000332.1	AdipoR1	*Oreochromis tanganicae*	GI
GenBank	VVHC01036010.1	AdipoR2	*Oreochromis tanganicae*	GI
GenBank	CAKLIZ010000012.1	AdipoR1	*Oreochromis mossambicus*	GI
GenBank	CAKLIZ010000026.1	AdipoR2	*Oreochromis mossambicus*	GI
GenBank	CM080751.1	AdipoR1	*Parachromis managuensis*	GI
GenBank	CM080770.1	AdipoR2	*Parachromis managuensis*	GI
GenBank	XM_005739511.1	AdipoR1	*Pundamilia nyererei*	GI
GenBank	XM_005725290.2	AdipoR2	*Pundamilia nyererei*	GI
GenBank	XM_004545016.2	AdipoR1	*Maylandia zebra*	GI
GenBank	XM_004548193.5	AdipoR2	*Maylandia zebra*	GI
GenBank	XM_026168257.1	AdipoR1	*Astatotilapia calliptera*	GI
GenBank	XM_026147387.1	AdipoR2	*Astatotilapia calliptera*	GI
GenBank	NM_001327754.1	AdipoR1a	*Danio rerio*	GI
GenBank	NM_213500.1	AdipoR1b	*Danio rerio*	GI
GenBank	NM_001025506.2	AdipoR2	*Danio rerio*	GI
GenBank	XM_043252650.1	AdipoR1a	*Puntigrus tetrazona*	GI
GenBank	XM_043247714.1	AdipoR1b	*Puntigrus tetrazona*	GI
GenBank	XM_043236806.1	AdipoR2	*Puntigrus tetrazona*	GI
GenBank	XM_043225776.1	ADPN	*Puntigrus tetrazona*	GI
GenBank	XM_043260193.1	ADPN	*Puntigrus tetrazona*	GI
GenBank	XM_042762394.1	AdipoR1	*Cyprinus carpio*	GI
GenBank	XM_042734146.1	AdipoR1	*Cyprinus carpio*	GI
GenBank	XM_019112176.2	AdipoR1	*Cyprinus carpio*	GI

GenBank	XM_019108773.2	AdipoR1b	*Cyprinus carpio*	GI
GenBank	XM_019080673.2	AdipoR2	*Cyprinus carpio*	GI
GenBank	XM_042721747.1	AdipoR2	*Cyprinus carpio*	GI
GenBank	XM_019083697.2	ADPNa	*Cyprinus carpio*	GI
GenBank	XM_019106129.2	ADPNb	*Cyprinus carpio*	GI
GenBank	XM_019097827.2	ADPN	*Cyprinus carpio*	GI
GenBank	XM_042719168.1	ADPN	*Cyprinus carpio*	GI
GenBank	XM_039685158.1	ADPNa	*Pimephales promelas*	GI
GenBank	XM_039649621.1	ADPNb	*Pimephales promelas*	GI
GenBank	XM_039674055.1	AdipoR1a	*Pimephales promelas*	GI
GenBank	XM_039679258.1	AdipoR1b	*Pimephales promelas*	GI
GenBank	XM_039667785.1	AdipoR2	*Pimephales promelas*	GI
GenBank	XM_029690180.1	ADPNa	*Salmo trutta*	GI
GenBank	XM_029704091.1	ADPNb	*Salmo trutta*	GI
GenBank	LOC115169267	ADPN*	*Salmo trutta*	GI
GenBank	XM_029775655.1	AdipoR1a	*Salmo trutta*	GI
GenBank	XM_029693721.1	AdipoR1b	*Salmo trutta*	GI
GenBank	XM_029748495.1	AdipoR2	*Salmo trutta*	GI
GenBank	XM_036935031.1	ADPNa	*Oncorhynchus mykiss*	GI
GenBank	XM_021611257.2	ADPNb	*Oncorhynchus mykiss*	GI
GenBank	XM_021581491.2	ADPN	*Oncorhynchus mykiss*	GI
GenBank	XM_036985502.1	ADPN	*Oncorhynchus mykiss*	GI
GenBank	LOC110508240	ADPN*	*Oncorhynchus mykiss*	GI
GenBank	NM_001281345.1	AdipoR1a	*Oncorhynchus mykiss*	GI
GenBank	XM_021610144.2	AdipoR1	*Oncorhynchus mykiss*	GI
GenBank	NM_001281346.1	AdipoR2	*Oncorhynchus mykiss*	GI
GenBank	XM_036944957.1	AdipoR2	*Oncorhynchus mykiss*	GI
GenBank	XM_042068838.1	ADPNa	*Alosa sapidissima*	GI
GenBank	XM_042111070.1	ADPNb	*Alosa sapidissima*	GI
GenBank	XM_042088746.1	AdipoR1a	*Alosa sapidissima*	GI
GenBank	XM_042098025.1	AdipoR1	*Alosa sapidissima*	GI
GenBank	XM_042077880.1	AdipoR2	*Alosa sapidissima*	GI
GenBank	XM_042710237.1	ADPNa	*Clupea harengus*	GI
GenBank	XM_012835490.2	ADPNb	*Clupea harengus*	GI
GenBank	XM_031567932.2	AdipoR1a	*Clupea harengus*	GI
GenBank	XM_031566844.2	AdipoR1	*Clupea harengus*	GI
GenBank	XM_042705374.1	AdipoR1	*Clupea harengus*	GI
GenBank	XM_042710037.1	AdipoR2	*Clupea harengus*	GI
GenBank	XM_036554888.1	ADPNa	*Megalops cyprinoides*	GI
GenBank	XM_036522518.1	ADPN	*Megalops cyprinoides*	GI
GenBank	XM_036531060.1	AdipoR1a	*Megalops cyprinoides*	GI
GenBank	XM_036533729.1	AdipoR1	*Megalops cyprinoides*	GI
GenBank	XM_036517603.1	AdipoR2	*Megalops cyprinoides*	GI
GenBank	XM_018740994.2	ADPN	*Scleropages formosus*	GI
GenBank	XM_018753354.2	ADPN	*Scleropages formosus*	GI
GenBank	XM_018742153.1	AdipoR1	*Scleropages formosus*	GI

GenBank	XM_018730090.2	AdipoR2	*Scleropages formosus*	GI
GenBank	XM_035421358.1	ADPN	*Anguilla anguilla*	GI
GenBank	XM_035389347.1	AdipoR1a	*Anguilla anguilla*	GI
GenBank	XM_035382522.1	AdipoR1	*Anguilla anguilla*	GI
GenBank	XM_035425904.1	AdipoR2	*Anguilla anguilla*	GI
GenBank	XM_030767573.1	ADPNa	*Chanos chanos*	GI
GenBank	XM_030774755.1	ADPNb	*Chanos chanos*	GI
GenBank	XM_030777458.1	AdipoR1a	*Chanos chanos*	GI
GenBank	XM_030784680.1	AdipoR1b	*Chanos chanos*	GI
GenBank	XM_030783913.1	AdipoR2	*Chanos chanos*	GI
GenBank	XM_034038198.3	ADPN	*Acipenser ruthenus*	GI
GenBank	XM_034028455.3	ADPN	*Acipenser ruthenus*	GI
GenBank	XM_059027980.1	ADPN	*Acipenser ruthenus*	GI
GenBank	XM_034052255.3	AdipoR1	*Acipenser ruthenus*	GI
GenBank	XM_034007041.3	AdipoR1	*Acipenser ruthenus*	GI
GenBank	XM_059003425.1	AdipoR1	*Acipenser ruthenus*	GI
GenBank	XM_058986429.1	AdipoR2	*Acipenser ruthenus*	GI
GenBank	XM_034026757.3	AdipoR2	*Acipenser ruthenus*	GI

Note:

MSA: multiple sequence alignment; PA: phylogenetic analysis; 3D: 3D structure analysis; DP: domain prediction; GI: gene identification; * represent pseudogene.

Appendix Table S2. Information and sequence of the primers used in present study

Accession No.	Primer name	Primer sequence
KJ126772.1	RT-On-β-actin	Forward: CGGAATCCACGAAACCACCTA
		Reverse: CCAGACGGAGTATTTACGCTCA
NM_001287402.1	RT-On-IFN-γ	Forward: GGGTGGTGTTTTGGAGTCGT
		Reverse: GTAGCGAGCCTGAGTTGTTGGTG
AY428948.1	RT-On-TNF-α	Forward: CGTCGTCGTGGCTCTTTGTT
		Reverse: TGGGGCTCTGTTTTGTCGC
XM_019350387.2	RT-On-IL-6	Forward: GGAGACAGAGGAGGCGGAGAT
		Reverse: GAGTAGATGAGCAGACCCTTGGC
XM_025906636.1	RT-On-Perforin A	Forward: TTCTCCGACAGGAAGACCAAG
		Reverse: TGTAGTCACTGATGGCGTCTC
XM_003439678.4	RT-On-Granzyme B	Forward: GAGCATTTTGTGGTGACTGCTGC
		Reverse: CACGGTCTGCGAGAGGAATAGGT
XM_003452322.5	RT-On-CaM	Forward: TACCAAAGAGCTGGGCACTG
		Reverse: TCAGCAGCACTGATGTAGCC
ENSONIT00000028270.2	RT-On-IL-2	Forward: ATGTCGAGACCCAGGGAAAC
		Reverse: CAGGCCACAGGTGACAGTTA
XM_019360337.1	RT-On-CD122	Forward: GGAGGTGTGTTTGGCAAGTT
		Reverse: CTGAGTCAGGATGAGGGCAT
NSONIT00000018373	RT-On-CD44	Forward: GCATCGCCATCCTCAGACA
		Reverse: CCAGCATTTTCATCATAGCAGTAGAC
XP_005451408.1	RT-On-CaMKKβ	Forward: ACGCTGTATTGCTTCGTCTTTG
		Reverse: TGGGTGTACCTTAATCTGTGGG
XM_005471854.3	RT-On-CTRP9	Forward: CACAGCAAGAAACCCAAAGT
		Reverse: GCCCAGTAGGTCCAAGTTCA
XM_003441499.4	RT-On-AdipoR1	Forward: TGTTGAGCTGATGGAGCTGG
		Reverse: CGTTGTCCTTCAGCCACTCT
FJ914655.1	RT-On-Glut1	Forward: TCAACAAGAACGAAGAGAACAAGG
		Reverse: GGCTCCAATGGTGGCATAAAT
XM_003437654.5	RT-On-PKM	Forward: CTTCCTCGCCCAGAAAATGA
		Reverse: GGATAGTCTCCCTTGGCAGTC
XM_003448146.4	RT-On-PFKP	Forward: AAAGGAGGTTCAATGTTGGGCA
		Reverse: CGTTGTTGGATACGGTGGCA
NM_001319868.1	RT-On-AMPKα1	Forward: TCAGGTTATCAGCACCCCTACA
		Reverse: TCCTTTTCATCCAACTTTCCATT
NM_001319869.1	RT-On-AMPKα2	Forward: TCATAAGCACACCGACAGATTTT
		Reverse: GTCCTCCACCCGTCCATTC
XM_003441301.5	RT-On-AMPKγ1	Forward: CGTTGTTATGACCTTGTGCCC
		Reverse: CCAATGGAGATTTGTAATAGCGAT
XM_005471854.3	siRNA-CTRP9-1	Forward: AGGAGAUAAUGGAGAUAGGTT
		Reverse: CCUAUCUCCAUUAUCUCCUTT
	siRNA-CTRP9-2	Forward: GCUUUCUCCGUAGGACUCATT
		Reverse: UGAGUCCUACGGAGAAAGCTT

	siRNA-CTRP9-3	Forward: GCUUACUUCUUCACGUACCTT<br>Reverse: GGUACGUGAAGAAGUAAGCTT
	control-siRNA	Forward: UUCUCCGAACGUGUCACGUTT<br>Reverse: ACGUGACACGUUCGGAGAATT
NM_007393.5	RT-Mus-β-actin	Forward: GTCGTACCACAGGCATTGTGATGG<br>Reverse: GCAATGCCTGGGTACATGGTGG
NM_011400.4	RT-Mus-Glut1	Forward: TTGGTTCCTTCTCTGTCGGC<br>Reverse: ACATGGGCACAAAGCCAGTA
XM_036165880.1	RT-Mus-HK2	Forward: TTACCGTCTGGCTGACCAAC<br>Reverse: GCTCAGACCCTGCTCCATTT
NM_001253883.2	RT-Mus-PKM	Forward: TTAGGCCAGCAACGCTTGTA<br>Reverse: CTGCCGCCCTTCTGTGATAA
NM_019703.4	RT-Mus-PFKP	Forward: GAGGAAGGCGTTTTGAGGGA<br>Reverse: TTGCAAACATCTTGTGGCCG
NM_001306069.1	RT-Mus-AdipoR1	Forward: CAATGGGGCTCCTTCTGGTA<br>Reverse: AACGTCCCTCCCAGACCTTA
XM_005471854.3	R-On-CTRP9	Forward: GGAATTCATGTTGAAGACAAGGTTTA GGATTG<br>Reverse: ACGCGTCGACAGCCTCAAAGATCAAA AACCCAG
NP_898998.2	R-Mus-CTRP9	Forward: CCCAAGCTTGCCAGGACACCTGCCG GCAAGG<br>Reverse: CCGCTCGAGTCAAGAGCTGCTGAACA GCA

Note:

RT: qPCR; R: Recombinant; On: *Oreochromis niloticus*; Mus: *Mus musculus*.
